# The Role of Vibrators in Women’s Pelvic Health: An Alluring Tool to Improve Physical, Sexual, and Mental Health

**DOI:** 10.1007/s00192-024-05775-7

**Published:** 2024-04-26

**Authors:** Alexandra Dubinskaya, Priya Kohli, Poone Shoureshi, Catherine Breese, Victoria Scott, Jennifer T. Anger, Karyn S. Eilber

**Affiliations:** 1 Department of Surgery, Division of Urology, Cedar-Sinai Medical Center, Los Angeles, CA USA; 2 Los Angeles Institute for Pelvic & Sexual Health, Beverly Hills, CA USA; 3 Keck School of Medicine of USC, 1975 Zonal Ave, Los Angeles, CA 90033 USA; 4 Samuel Oschin Comprehensive Cancer Institute, Cedars Sinai Medical Center, 8700 Beverly Blvd, Los Angeles, CA 90048 USA; 5 Department of Urology, Gender Affirming Surgery, Urologic Reconstruction, and Female Pelvic Medicine, University of California San Diego, La Jolla, CA USA

**Keywords:** Female sexual function, Pain, Pelvic organ prolapse, Urinary incontinence, Depression, Vibrator

## Abstract

**Introduction and hypothesis:**

In limited studies vibrators have been shown to improve sexual function and pelvic floor health; however, there are even fewer studies on the effect of vibrator use on overall genitourinary and mental health. To investigate the effect of regular vibrator use on sexual, genitourinary, and mental health in addition to quality of life.

**Methods:**

We performed a prospective pilot study of women aged 18 to 80 years recruited from a urogynecology clinic. Study participants were instructed to use a vibrator according to the protocol. Sexual function, pelvic floor function, mental health, and pelvic examination were assessed at the initial visit and at 3 months’ follow-up using validated questionnaires.

**Results:**

Of the 79 participants enrolled in the study, 53 women (66%) completed the study. The mean age of the participants was 54.7 years (range 19–80 years), and the majority of participants were white (*n* = 59, 74.7%), post-menopausal (*n* = 48, 60.8%), and not receiving systemic (*n* = 63, 79.7%) or local (*n* = 63, 79.7%) hormone therapy. Sexual function significantly improved over time (*p* = 0.002), whereas the rate of bothersome pelvic organ prolapse symptoms and pain scores significantly decreased (*p* = 0.034 and 0.0008 respectively). Rates of urge urinary incontinence decreased although this was not statistically significant (*p* = 0.059). There was a significant improvement in the gross appearance of lichen sclerosus lesions (*p* = 0.025) and in the severity of vaginal atrophy (*p* = 0.018). Rates of depression were significantly decreased (*p* = 0.011).

**Conclusions:**

Vibrator use was associated with improved sexual, genitourinary, and mental health.

**Supplementary information:**

The online version contains supplementary material available at 10.1007/s00192-024-05775-7

## Introduction

Throughout a woman’s life, her body, especially the genitourinary system, undergoes several changes. Changes in a woman’s pelvic floor start during puberty and are significantly affected by childbirth, with unavoidable trauma to the pelvic floor muscles and connective tissue, such as injury to the urinary and anal sphincters, as well as neuropathy from prolonged pushing. Aging and menopause can affect the pelvic floor. Hormonal fluctuations combined with progressive weakening of the connective tissue over time often result in pelvic floor disorders such as pelvic organ prolapse and incontinence. Furthermore, many women experience symptoms of genitourinary syndrome of menopause (GSM), including loss of natural vaginal lubrication and a decrease in vaginal caliber, resulting in dyspareunia and impaired sexual function. Although pelvic floor physical therapy, devices such as pessaries, and surgery can improve these conditions, a single modality that could promote and support a woman’s pelvic, sexual, and overall health would be ideal.

One such modality is a vibrator. Studies have shown that perineal vibratory stimulation can improve urinary incontinence [1, 2] and sexual function [3–11], and vibrators are accepted as a health tool by medical providers [12]. All this suggests that a vibrator may be the best device for female pelvic rehabilitation, similar to the concept of penile rehabilitation post-prostatectomy [13]. The objective of our study was to investigate the effects of regular vibrator use on sexual, genitourinary, and mental health, in addition to quality of life, in a population of women seeking care at a urogynecology clinic.

## Materials and Methods

This Institutional Review Board-approved prospective pilot study was conducted at a urogynecology clinic in a tertiary care hospital in metropolitan Los Angeles between April 2021 and December 2022.

English-speaking cisgender women aged 18 to 80 years with sexual dysfunction, pelvic floor disorder, and/or genitourinary syndrome of menopause were recruited for participation in the urogynecological clinic via direct referrals, intra-organizational email communication, and social media. Exclusion criteria included women who were non-English speaking, not assigned female at birth, had a history of genital gender-affirming surgeries, had a history of genital mutilation, were currently pregnant, within 6 months postpartum, or who had medical or mental health conditions affecting their dexterity and ability to operate the vibrator.

Standardized screening interviews were conducted either in person or via phone by research personnel. Screening interviews included questions about gender, the presence of any pelvic floor disorders, sexual dysfunction, and interest in participation in the study. Conditions categorized as sexual dysfunction in this study included hypoactive sexual desire disorder, decreased arousal, decreased lubrication, and absent or muted orgasm. The conditions included in the pelvic floor disorders were stress urinary incontinence, urge urinary incontinence, pelvic organ prolapse, interstitial cystitis, pelvic floor dysfunction (hypertonic pelvic floor), genitourinary syndrome of menopause, and skin dermatoses.

After informed consent was signed, demographic information was obtained, including age, menopausal status, hormone therapy history, medical history, and sexual history. Sexual function was assessed using the Female Sexual Function Index (FSFI) and the Pelvic Organ Prolapse/Urinary Incontinence Sexual Questionnaire IUGA-Revised (PISQ-IR). The FSFI is a validated 19-item measure of sexual function in four domains: desire, arousal, orgasm, and sexual pain. The total score ranges from 0 to 36, with higher scores indicating better sexual functioning. A total score below 26.6 indicates clinical Female Sexual Dysfunction (FSD). The PISQ-IR is a validated measure of sexual function among sexually active women with and without a partner. This questionnaire contains 20 questions and is used to assess sexual activity in women with urinary incontinence and pelvic organ prolapse. Higher scores indicated better sexual function.

Pelvic floor function was assessed with the following validated questionnaires: Pelvic Floor Disability Index, Female Genitourinary Pain Index (F-GUPI), and the Interstitial Cystitis Symptoms Index/Interstitial Cystitis Problem Index (ICSI-ICPI). The Pelvic Floor Distress Inventory (PFDI) is a condition-specific quality-of-life questionnaire for women with bowel, bladder, and/or pelvic symptoms. The scores ranged between 0 and 300, with higher scores indicating more severe distress. The F-GUPI is used to assess symptom severity and impact in women with genitourinary pain complaints. The score ranges from 0 to 45, and is the sum of the individual scores for the domains of pain, urinary symptoms, and impact on quality of life. The ICSI-ICPI is the sum of four items that measure urinary urgency, frequency, night-time urination, and pain/burning. In addition, participants were asked if they had a prolapse and the degree of bother it caused. Responses from questionnaires, physical examinations, and subjective reports were all recorded and assessed at the initial appointment and follow-up.

Mental health and quality of life were assessed using the Patient Health Questionnaire (PHQ9) and the Health-Related Quality of Life-Short Form (SF12) respectively. The PHQ9 is a validated tool for the assessment of depression. Depression severity was assessed by summarizing the scores assigned to each category. Scores range between 1 and 27, with higher scores correlating with more severe depression. The SF12 is a general health measure that assesses the impact of health on everyday life. The SF12 consists of eight domains and generates two separate summary scores, physical functional scores (PCS), and mental function scores (MCS), each ranging from 0 to 100. Higher scores indicate better health-related quality of life.

A baseline pelvic examination was performed to evaluate vulvar dermatoses, vaginal epithelium changes, pelvic organ prolapse, urinary or anal incontinence, pelvic floor muscle strength, and trigger points. The presence and severity of vaginal atrophy were recorded based on examiner assessment on a scale of 1 to 3, corresponding to mild, moderate, and severe changes respectively. This classification was based on the degree of anatomical changes in the genitalia that have been routinely used in our practice, as no validated tool has been developed to date. The assessment included the color and friability of the tissue, the amount of vaginal lubrication, the presence of a urethral caruncle, the presence of rugae, elasticity, vaginal caliber, resorption or fusion of the labia majora and/or minora, and introital retraction [14]. Similarly, the presence and severity of lichen sclerosus were recorded based on examiner assessment on a scale of 1 to 3, corresponding to mild, moderate, and severe respectively. This classification was based on the amount of involved skin, as no validated tools have been developed to date, and it has been routinely used in our practice. The assessment of vaginal tissue assessment was performed by the same examiner. Pelvic organ prolapse was assessed by the examiner and categorized based on the Baden–Walker Halfway Scoring System. To mitigate potential bias, all assessment measurements were recorded during the examination, and the investigator was not allowed to review the initial examination findings prior to seeing the study participants at their 3-month follow-up visit.

**Fig. 1:**
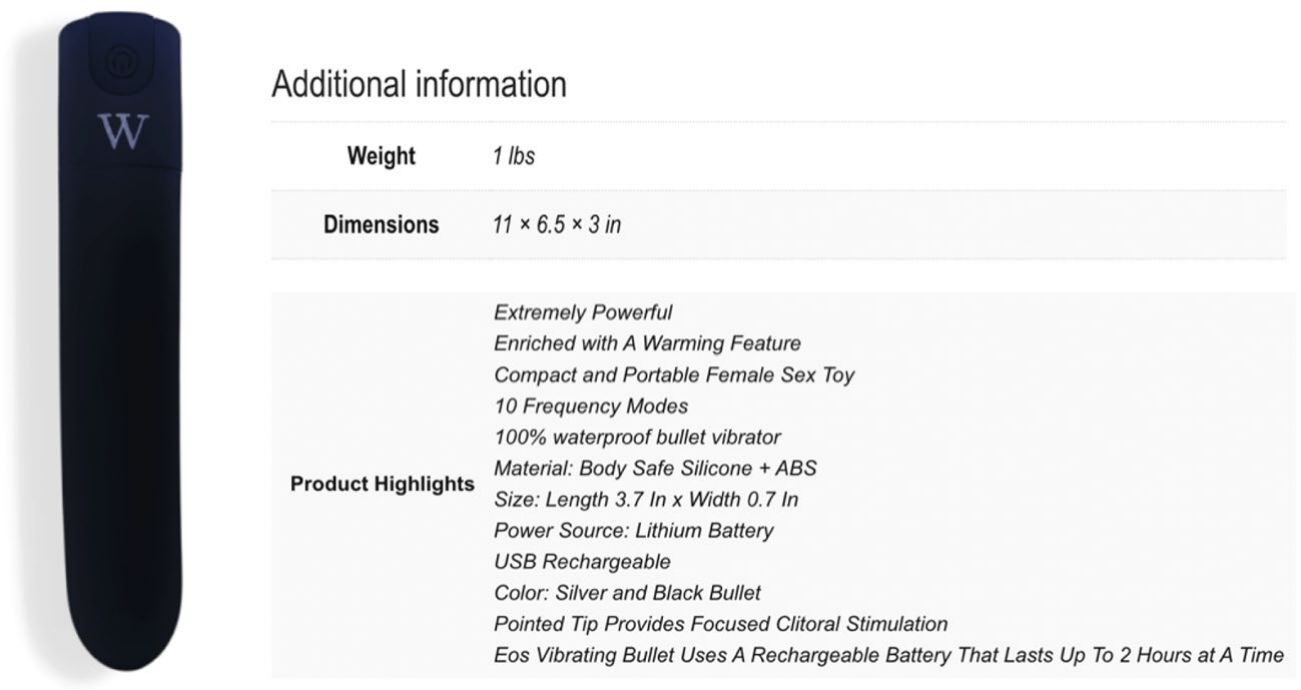
Bullet vibrator

After baseline data collection, women were given a multimodal bullet vibrator (Fig. 1) and were instructed to apply it to their external genitalia for 5–10 min, two to three times per week for 3 months. Participants were encouraged to focus on adhering to the protocol rather than on reaching an orgasm. They were also advised to use a vibrator to determine whether they felt sexual desire or interest. Setting reminder alarms on their smartphones is highly encouraged. Each woman was given a calendar diary to track her vibrator use and any additional sexual encounter. Participants were encouraged to use the vibrator alone and with their partners. Instruction on safe use and cleaning was discussed and provided in a written form. Women who had never masturbated or were not familiar with their anatomy were educated by research personnel and recommended to visit the Betty Dodson website (https://www.dodsonandross.com/sexfeature/first-time-orgasm?utm_source=www.dodsonandross.com), as her method of achieving an orgasm has been evaluated and demonstrated to be effective [8]. Women were also given information on lubricants and their compatibility with the vibrator (Appendix 1, Supplemental Material). Women were not compensated for participation, but were allowed to keep the vibrator after the completion of the study.

The primary outcomes of this pilot study were the effects of regular vibrator use on sexual function, pelvic floor disorders/genitourinary function, and mental health, including health-related quality of life. Secondary outcomes were the identification of any demographic or health-related differences between the participants who completed the study and those who dropped out.

Changes in pelvic floor disorders/genitourinary health, sexual function, and mental health from baseline to the 3-month follow-up were tested using a McNemar test for dichotomous data, a paired *t* test for normally distributed data, or a Wilcoxon sign test for rank data. Statistical analysis was performed using SAS v9.4, and statistical significance was set at *p* < 0.05. Differences in demographics and questionnaire-based data between participants who completed the study and those who dropped out were tested using a Chi-squared test for categorical data, a two-sample *t* test for normally distributed data, or a Wilcoxon rank sum test, as appropriate. This study was designed as a pilot study to collect data for future in-depth investigations; therefore, no power analysis was performed.

## Results

The demographics of the study population are presented in Table 1. A total of 79 participants consented to participate in the study, and 53 (66%) completed the study. The average age of the participants was 54.7 years (range, 19–80 years). The majority of women were white (*n* = 59, 74.7%) and postmenopausal (*n* = 48, 60.8%), and did not receive either systemic or local hormone therapy (*n* = 63, 79.7%). The majority of the participants had at least a college degree (*n* = 68, 86%). Among the participants who completed the study, the frequency of solo sexual activity and vibrator use increased, whereas pain due to any type of sexual activity decreased (Table 2). Four patients experienced issues with vibrator malfunction, and the devices were replaced, allowing the participants to continue with the study. The most common reasons for dropping out were insufficient time to use the vibrator, medical conditions (breast cancer, interstitial cystitis flare, mental health), and partner-related concerns (partner’s medical conditions).

**Table 1:** General demographics at enrollment for the full cohort, along with differences between participants who completed the study versus those who dropped out

Characteristics	All subjects (*N* = 79)	Completers (*N* = 53)	Drop-outs (*N* = 26)		*p* value	
Age (years)	54.7 ± 15.8	53.7 ± 16.4	56.9 ± 14.6		0.392*	
Race						
White	59 (75)	40 (76)	19 (73)		0.435**	
Hispanic	11 (14)	8 (15)	3 (12)			
Black	7 (9)	3 (6)	4 (15)			
Asian	2 (3)	2 (4)	0 (0)			
Parity	1.2 ± 1.2	1.3 ± 1.2	1.0 ± 1.1		0.259***	
Menopausal status						
Premenopausal	25 (32)	19 (36)	6 (23)		0.306**	
Perimenopausal	6 (8)	5 (9)	1 (4)			
Postmenopausal	48 (61)	29 (55)	19 (73)			
Hormonal therapy						
HRT	16 (20)	9 (17)	7 (27)		0.374**	
Vaginal estrogens	16 (20)	12 (23)	4 (15)		0.559**	
Hormonal contraception	10 (13)	8 (15)	2 (8)		0.484**	
Comorbidities						
HTN	10 (13)	8 (15)	2 (8)		0.484**	
Diabetes	3 (4)	2 (4)	1 (4)		> 0.999**	
Neurological conditions	10 (13)	8 (15)	2 (8)		0.483**	
Depression	27 (34)	17 (32)	10 (39)		0.619**	
Anxiety	22 (28)	12 (23)	10 (39)		0.183**	
Sexual activity						
Partnered	42 (53)	32 (60)	10 (39)		0.093**	
Solo	56 (71)	36 (68)	20 (77)		0.444**	
Vibrator use	39 (49)	25 (47)	14 (54)		0.637**	
Penetration	51 (65)	39 (74)	12 (46)		0.024**	
Pain	36 (46)	25 (48)	11 (42)		0.810**	
FSFI score						
Total	19.1 ± 9.2	16.9 ± 9.6	19.7 ± 9.0		0.274*	
Desire	3.1 ± 1.3	2.9 ± 1.1	3.1 ± 1.3		0.256*	
Arousal	3.4 ± 1.9	3.0 ± 2.1	3.5 ± 1.9		0.359*	
Lubrication	3.3 ± 2.2	3.4 ± 2.4	3.3 ± 2.1		0.874*	
Orgasm	3.6 ± 2.1	3.4 ± 2.4	3.7 ± 2.1		0.595*	
Satisfaction	2.6 ± 1.8	1.7 ± 1.4	2.9 ± 1.8		0.015*	
Pain	2.8 ± 2.4	2.5 ± 2.6	2.9 ± 2.3		0.595*	
SF12 PCS	47.3 ± 10.3	52.0 ± 7.7	45.8 ± 10.7		0.042*	
Pelvic health measures						
Pelvic organ prolapse (objective)	23 (29)	17 (32)	6 (23)		0.444**	
Pelvic organ prolapse bother (subjective)	14 (18)	12 (23)	2 (8)		0.205**	
Urge urinary incontinence	27 (34)	16 (30)	11 (42)		0.320**	
Stress urinary incontinence	31 (39)	19 (34)	12 (46)		0.464**	
Mixed urinary incontinence	12 (15)	7 (13)	5 (19)		0.516**	
Fecal incontinence	6 (8)	3 (6)	3 (12)		0.389**	
Kegels (Oxford scale)	3.1 ± 1.4	3.1 ± 1.4	3.0 ± 1.6		0.759***	
Lichen sclerosus	6 (8)	6 (11)	0 (0)		0.169**	
Vaginal atrophy	38 (50)	23 (43)	15 (65)		0.545**	

Data are presented as means ± SD or *n* (percentage)

*FSFI* Female Sexual Function Index, *SF12 PCS* Health-Related Quality of Life Questionnaire—Physical Function Scores

*Significance calculated by *t* test

**Significance calculated by Fisher’s exact test

***Significance calculated by Wilcoxon rank sum test

**Table 2:** Sexual function change

Sexual activity	Baseline, *n* (%)	Completion, *n* (%)	*p* value
Partnered sexual activity	32 (60.4)	36 (67.9)	0.16
Solo sexual activity	36 (67.9)	51 (96.2)	0.0001
Vibrator use	25 (47.2)	52 (98.1)	< 0.0001
Vaginal penetration	39 (73.6)	44 (83.0)	0.17
Pain with sexual activity	25 (48.1)	8 (15.1)	< 0.0001
Monthly sexual activity frequency	3.0 ± 3.6	3.5 ± 3.7	0.26
Monthly vibrator use frequency	2.8 ± 3.1	8.2 ± 2.9	< 0.0001

Data are presented as *n* (percentage) or means ± SD

### Effects of Vibrator Use on Sexual Health

**Fig. 2:**
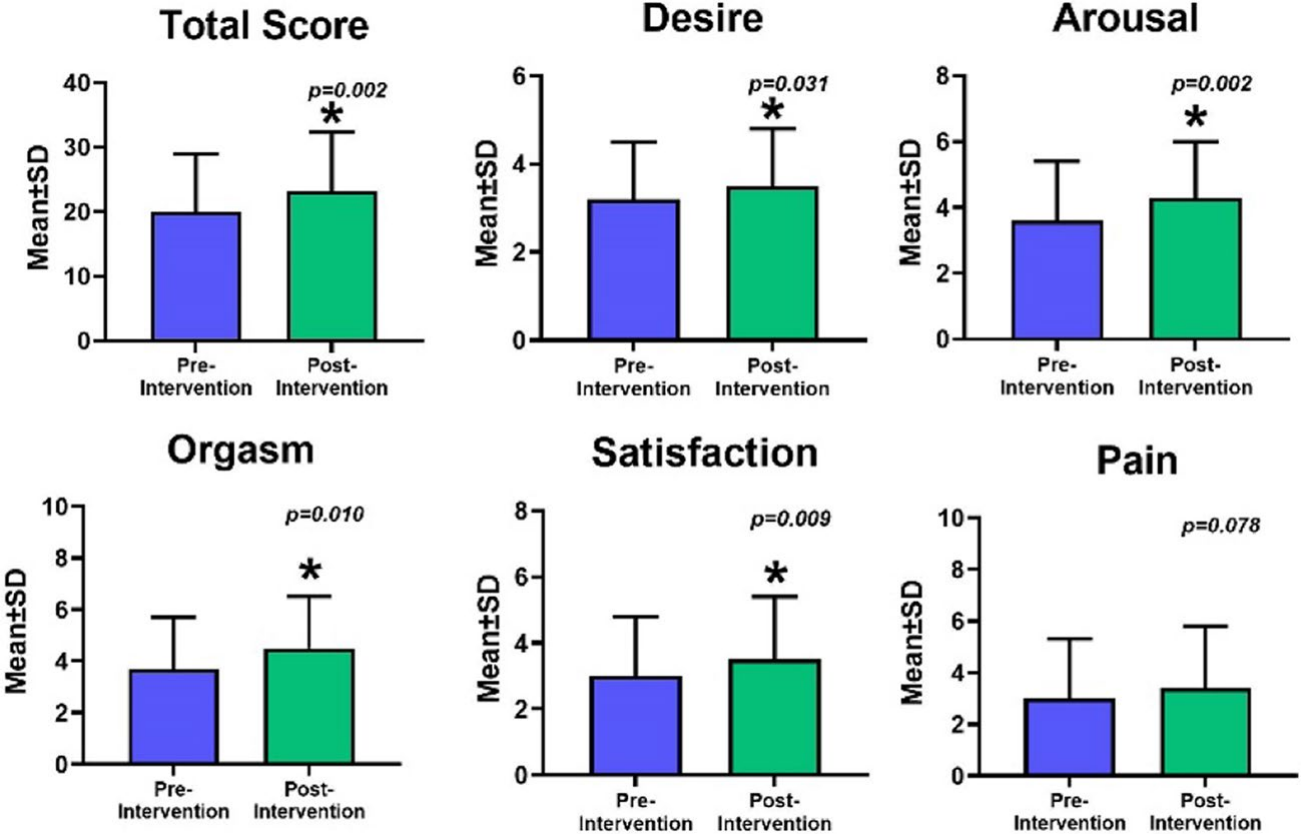
Changes in Sexual function based on Female Sexual Function Index scores

At 3 months’ follow-up, there were significant increases in FSFI scores over time, including the total score (*p* = 0.002), domains of desire (*p* = 0.031), arousal (*p* = 0.002), orgasm (*p* = 0.010), and satisfaction (*p* = 0.009), as shown in Fig. 2.

Initially, we included the PISQ-IR questionnaire, which was designed to assess solo sexual function, but the questionnaire was removed owing to the high proportion of missing data.

### Effects of Vibrator Use on Genitourinary Health

**Fig. 3:**
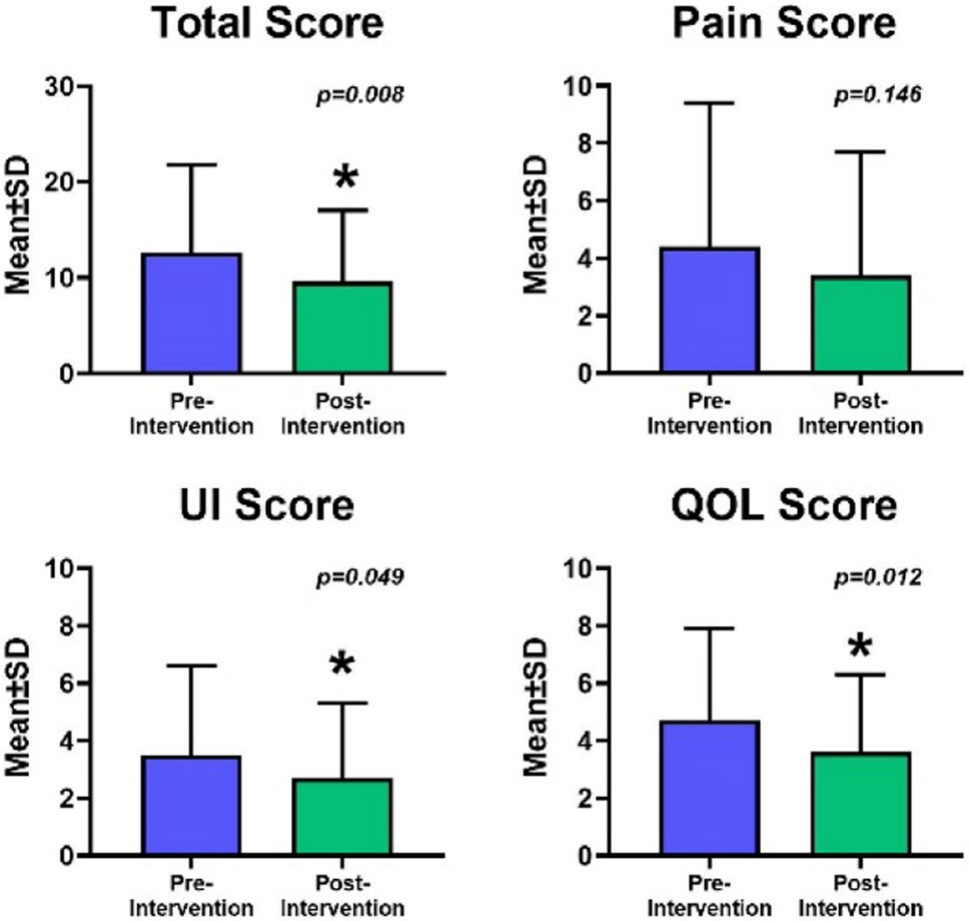
Changes in Genitourinary Pain Index scores. *UI* urinary incontinence, *QOL* quality of life

Although there was no difference in the rate of objective measurements of pelvic organ prolapse over time (32.1% vs 24.5%, *p* = 0.157), the rate of bothersome pelvic organ prolapse symptoms significantly decreased (22.6% vs 11.3%, *p* = 0.034). Rates of urge urinary incontinence decreased slightly, but this was not statistically significant (30.2% vs 20.8%, *p* = 0.059). No changes were observed in the rates of stress urinary incontinence or fecal incontinence. However, there was a significant improvement in the appearance of lichen sclerosus lesions (11.3% vs 2%, *p* = 0.025) and the severity of vaginal atrophy (*p* = 0.018; Table 3). There were no changes in PFDI-20 scores (*p* = 0.113) or ICSI/ICPI scores (*p* = 0.282, *p* = 0.137 respectively) from baseline to study completion, but GUPI scores improved (*p* = 0.008; Fig. 3).

**Table 3:** Pelvic health outcomes

Findings	Baseline	Completion	*p* value
Pelvic organ prolapse (objective)	17 (32.1)	13 (24.5)	0.16
Pelvic organ prolapse—bother (subjective)	12 (22.6)	6 (11.3)	0.0339
Urge urinary incontinence	16 (30.2)	11 (20.8)	0.0588
Stress urinary incontinence	19 (35.8)	15 (28.3)	0.10
Mixed urinary incontinence	7 (13.2)	5 (9.4)	0.16
Fecal incontinence	3 (5.7)	1 (1.9)	0.16
Kegels (strength on Oxford scale)	3.1 (0–5)	3.1 (0–5)	0.99
Lichen sclerosus (number with skin lesions present)	6 (11.3)	1 (2.0)	0.0253
Vaginal atrophy (presence overall)	23 (43.4)	22 (43.1)	0.07
Severity 1	6 (11.3)	14 (27.5)	
Severity 2	12 (22.6)	6 (11.8)	0.0176
Severity 3	5 (9.4)	2 (3.9)	

Data are presented as *n* (percentage) except for Kegels

### Effects of Vibrator Use on Mental Health and Quality of Life

**Fig. 4:**
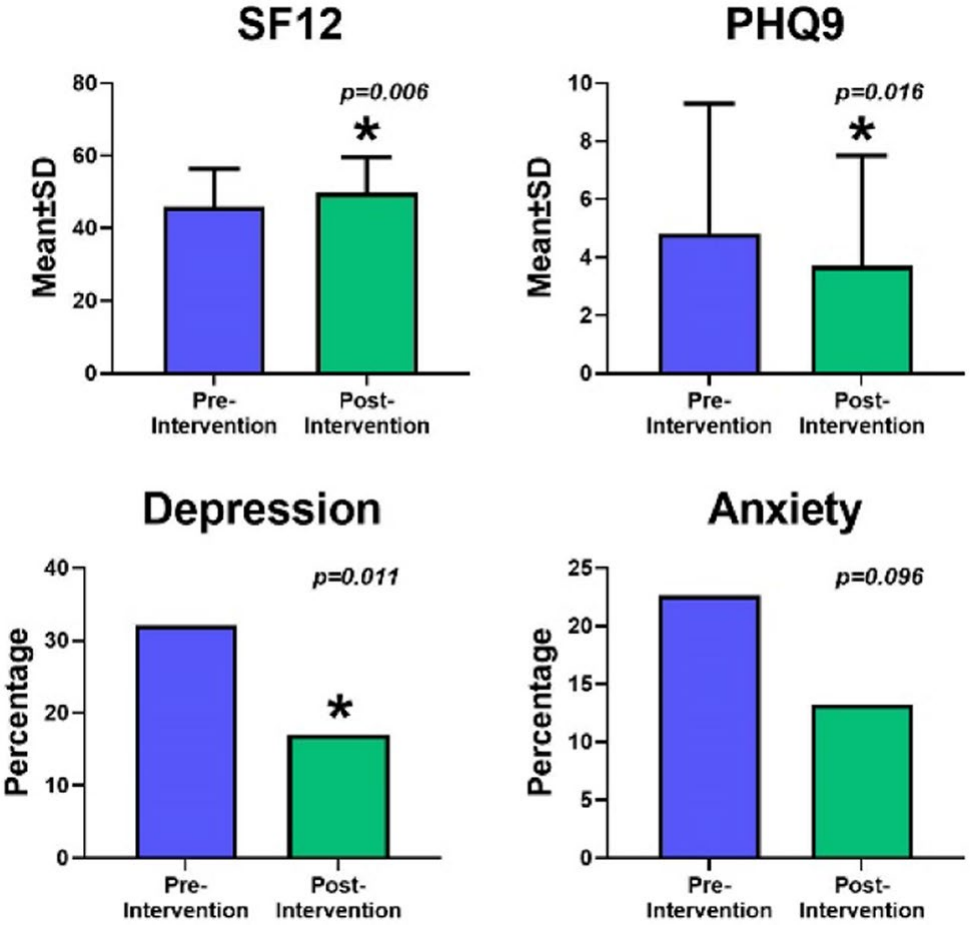
Mental health and quality of life outcomes. *SF12* Health-Related Quality of Life-Short Form, *PHQ9* Patient Health Questionnaire

The rate of subjective depression significantly decreased (*p* = 0.011, Fig. 4), as did the overall presence of subjectively assessed mental health issues (*p* = 0.021). Health-related quality of life significantly improved at the end of the study (*p* = 0.006). There were no significant changes in subjective anxiety rate (*p* = 0.096).

### Differences Between Participants Who Completed the Study Versus those Who Dropped Out

Patients who completed the study were more likely to have vaginal penetration during sexual activity (74% vs 46%, *p* = 0.017; Table 1). There was a trend toward more partnered sexual activity among those who completed the study (60% vs 39%, *p* = 0.067). Completers were more likely to have higher FSFI satisfaction subscale scores (*p* = 0.015) and lower overall SF12 PCS at enrollment (*p* = 0.042), which correlated with lower health-related quality of life. No differences were observed between completers and noncompleters in terms of the examination findings at enrollment.

## Discussion

Our study demonstrated that regular vibrator use has several benefits. In the area of sexual function, there was significant improvement in multiple domains such as desire, arousal, orgasm, and satisfaction. These findings are consistent with those in the existing literature. It has been shown that vibratory stimulation improves pelvic circulation by increasing genital blood flow [3–5] and plays an important role in local arousal by increasing vaginal perfusion and enhancing vaginal lubrication [6]. Furthermore, vibrators have been studied as a treatment for primary anorgasmia and delayed orgasm with successful results [7–9]. Vibrators generate vasocongestion sensitivity in genitalia thereby giving women the ability to experience multiple orgasms [3, 4]. Moreover, studies done in men demonstrated that local vibration applied to the external genitalia stimulates superficial and deep nerve receptors that transmit stimuli to the spinal cord, causing changes in sexual physiology [4, 9, 15]. Additionally, population-based studies have shown that vibrators are considered an accepted modality for enhancing a woman’s sexual experience [10, 11], as well as a health tool distributed by medical providers [12].

Participants who engaged in partnered sexual activity had a higher rate of study completion in addition to significantly improved sexual function outcomes. The role of a partner in adherence to therapy cannot be determined by our study; however, having a partner can potentially make participants more goal oriented and promote feelings of encouragement to continue the intervention. Additionally, a partner can be a “litmus test,” which helps to recognize any improvements in sexual functioning.

In the area of pelvic floor function, subjectively reported bothersome symptoms of pelvic organ prolapse significantly improved, whereas physical examination findings did not change significantly. Prior studies showed improved muscle strength, which reduced urinary incontinence, and thereby improved the overall quality of life with regular vibrator use [1, 2]. Although the presence of vaginal atrophy remained the same, the severity of atrophic changes improved significantly. One explanation for these improved symptoms may be the positive impact of increased blood flow from arousal that occurs with the use of a vibrator. Why the symptoms of prolapse were reduced is a bit less clear, although improved vaginal atrophy (possibly from increased blood flow) could explain the reduction in prolapse symptoms. Increased blood flow may also be responsible for the significant improvement in the appearance of lichen sclerosus lesions.

Urinary incontinence outcomes did not reach statistical significance; however, a positive trend was observed. It is important to point out that in our study, women were instructed to apply a vibrator to their genital area without inserting it vaginally. It is possible that the application area plays a role in the outcomes.

This study demonstrated significant improvement in depression and health-related quality of life. This is similar to Skałacka et al., who demonstrated that global life satisfaction improved among adults who engaged in subtle sexual activities, including masturbation [16]. This again reinforces the importance of sexual health as a part of overall health and well-being. We did not control for possible confounders, such as time of year and life stressors. All participants were encouraged to continue taking their regular medications.

All these positive effects of vibrator use—improvement in sexual function and pelvic floor disorders, quality-of-life improvement with varied sexual activity, and its acceptance from medical health care providers—suggest that the ideal device for pelvic rehabilitation might be a vibrator.

This study has some limitations. First, the study did not have a control group or randomization; however, this was a pilot study for future research planning. Second, there were variations in vibrator use among the participants. The duration of each vibrator use session was based on self-reporting, with the possibility of bias. In addition, there may be variations in the vibrator application and its settings. Another limitation is the assessment of subjective and objective outcomes in women with female sexual dysfunction. Traditionally, sexual function is assessed via validated questionnaires, such as the FSFI; however, this questionnaire primarily targets women in partnered sexual activity with penetrative vaginal intercourse. There is a growing need to develop and validate a questionnaire to assess sexual function among cisgender women engaging in solo sexual activities. Having such a tool will allow us to better assess treatment interventions in both partnered and nonpartnered women. Furthermore, there may be a potential selection bias, as the results were evaluated solely in women who completed the study, lacking detailed information on those who did not participate.

Although there are limitations, these were balanced by the significant strengths of this study. This was a prospective study using a novel approach that included a quantitative assessment of the effect of the regular use of vibrators. Furthermore, despite the lack of financial compensation for participants, our study demonstrated an acceptable retention rate. Finally, this study demonstrated the feasibility of a potential alternative treatment and/or rehabilitation modality, and support for multiple conditions that are common among women at different stages of life.

## Conclusion

Our pilot study illustrated how vibrators can be an excellent tool for improving and maintaining sexual, pelvic, and overall health. Regular vibrator use can improve female sexual and genitourinary functions as well as mental health and quality of life. Therefore, consideration should be given to recommending vibrators as rehabilitation tools to improve overall health.

### Supplementary information

Below is the link to the electronic supplementary material.

Supplementary file1 (DOCX 13 KB)

## Data Availability

The data that support the findings of this study are available from the corresponding author upon reasonable request.

## References

[1] Rodrigues MP, Paiva LL, Ramos JGL, Ferla L (2018). Vibratory perineal stimulation for the treatment of female stress urinary incontinence: a systematic review. Int Urogynecol J.

[2] Dubinskaya A, Horwitz R, Scott V, Anger J, Eilber K (2023). Is it time for doctors to Rx vibrators? A systematic review of pelvic floor outcomes. Sex Med Rev.

[3] Amberson JI, Hoon PW (1985). Hemodynamics of sequential orgasm. Arch Sex Behav.

[4] Rullo JE, Lorenz T, Ziegelmann MJ, Meihofer L, Herbenick D, Faubion SS (2018). Genital vibration for sexual function and enhancement: a review of evidence. Sex Relation Ther.

[5] Sønksen J, Ohl DA, Bonde B, Laessøe L, McGuire EJ (2007). Transcutaneous mechanical nerve stimulation using perineal vibration: a novel method for the treatment of female stress urinary incontinence. J Urol.

[6] Dubinskaya A, Guthrie T, Anger JT (2021). Local genital arousal: mechanisms for vaginal lubrication. Curr Sex Health Rep.

[7] Graham CA, Binik YM, Hall KSK (2014). Orgasm disorders in women. Principles and practice of sex therapy.

[8] Struck P, Ventegodt S (2008). Clinical holistic medicine: teaching orgasm for females with chronic anorgasmia using the Betty Dodson method. Sci World J.

[9] Nelson CJ, Ahmed A, Valenzuela R, Parker M, Mulhall JP (2007). Assessment of penile vibratory stimulation as a management strategy in men with secondary retarded orgasm. Urology.

[10] Esteve-Ríos A, Garcia-Sanjuan S, Oliver-Roig A, Cabañero-Martínez MJ (2020). Effectiveness of interventions aimed at improving the sexuality of women with multiple sclerosis: a systematic review. Clin Rehabil.

[11] Herbenick D, Reece M, Schick V (2011). Beliefs about women’s vibrator use: results from a nationally representative probability survey in the United States. J Sex Marital Ther.

[12] Zolnoun D, Lamvu G, Steege J (2008). Patient perceptions of vulvar vibration therapy for refractory vulvar pain. Sex Relation Ther.

[13] Fode M, Borre M, Ohl DA, Lichtbach J, Sønksen J (2014). Penile vibratory stimulation in the recovery of urinary continence and erectile function after nerve-sparing radical prostatectomy: a randomized, controlled trial. BJU Int.

[14] Bleibel B, Nguyen H. Vaginal atrophy. In: StatPearls. Treasure Island, FL: StatPearls. 2023. Available from: https://www.ncbi.nlm.nih.gov/books/NBK559297/. Accessed 12 Apr 2023.

[15] Steers WD (2000). Neural pathways and central sites involved in penile erection: neuroanatomy and clinical implications. Neurosci Biobehav Rev.

[16] Skałacka K, Gerymski R (2019). Sexual activity and life satisfaction in older adults. Psychogeriatrics.

