# Supplementary material for: The Role of Vibrators in Women’s Pelvic Health: An Alluring Tool to Improve Physical, Sexual, and Mental Health
Source: Int Urogynecol J. 2024 Apr 26;35(5):1085–92. doi: 10.1007/s00192-024-05775-7 (PMC11150285; doi:10.1007/s00192-024-05775-7)
Supplement: Supplementary file 1 — Supplementary file1 (DOCX 13 KB) [file 192_2024_5775_MOESM1_ESM.docx]

| Usage\ Type of Lube | Water – Based Lube | Oil – Based Lube | Silicone – Based Lube |
| --- | --- | --- | --- |
| Good for sex/masturbation | Yes | Yes | Yes |
| Good for actions in a water | No | Yes | Yes |
| Good for sensitive skin and vaginas | Yes | Yes | No |
| Good with silicone sex toys | Yes | No | No |
| Good with metal, glass, hard plastic toys | Yes | Yes | Yes |
| Good with condoms | Yes | No | Yes |

Appendix 1:

Different types of lubricants.
